# Provider attitudes about childhood tuberculosis prevention in Lesotho: a qualitative study

**DOI:** 10.1186/s12913-020-05324-0

**Published:** 2020-05-25

**Authors:** Yael Hirsch-Moverman, Joanne E. Mantell, Limakatso Lebelo, Andrea A. Howard, Anneke C. Hesseling, Sharon Nachman, Koen Frederix, Llang Bridget Maama, Wafaa M. El-Sadr

**Affiliations:** 1 ICAP at Columbia University, Mailman School of Public Health, 722 West 168th Street, MSPH Box 18, New York, NY 10032 USA; 2 Department of Epidemiology, Columbia University, New York, NY USA; 3 HIV Center for Clinical & Behavioral Studies, Division of Gender, Sexuality and Health, at the New York State Psychiatric Institute and Columbia University Irving Medical Center, Department of Psychiatry, New York, NY USA; 4 Desmond Tutu TB Centre, Department of Paediatrics and Child Health, Faculty of Medicine and Health Sciences, Stellenbosch University, Tygerberg, South Africa; 5 Pediatric Infectious Diseases, SUNY Stony Brook, Stony Brook, NY USA; 6 Lesotho Ministry of Health National Tuberculosis Program, Maseru, Lesotho

**Keywords:** Tuberculosis, Child TB contact management, Isoniazid preventive therapy, Qualitative evaluation

## Abstract

**Background:**

The World Health Organization estimated that 1.12 million children developed tuberculosis (TB) in 2018, and at least 200,000 children died from TB. Implementation of effective child contact management is an important strategy to prevent childhood TB but these practices often are not prioritized or implemented, particularly in low- and middle-income countries. This study aimed to explore attitudes of healthcare providers toward TB prevention and perceived facilitators and challenges to child contact management in Lesotho, a high TB burden country. Qualitative data were collected via group and individual in-depth interviews with 12 healthcare providers at five health facilities in one district and analyzed using a thematic framework.

**Results:**

Healthcare providers in our study were interested and committed to improve child TB contact management and identified facilitators and challenges to a successful childhood TB prevention program. Facilitators included: provider understanding of the importance of TB prevention and enhanced provider training on child TB contact management, with a particular focus on ruling out TB in children and addressing side effects. Challenges identified by providers were at multiple levels -- structural, clinic, and individual and included: [1] access to care, [2] supply-chain issues, [3] identification and screening of child contacts, and [4] adherence to isoniazid preventive therapy.

**Conclusions:**

Given the significant burden of TB morbidity and mortality in young children and the recent requirement by the WHO to report IPT initiation in child contacts, prioritization of child TB contact management is imperative and should include enhanced provider training on childhood TB and mentorship as well as strategies to eliminate challenges. Strategies that enable more efficient child TB contact management delivery include creating standardized tools that facilitate the implementation, tracking, and monitoring of child TB contact management coupled with guidance and mentorship from the district health management team. To tackle access to care challenges, we propose delivering intensive community health education, conducting community screening more efficiently using standardized tools, and facilitating access to services in the community.

## Background

The World Health Organization (WHO) estimated that 1.12 million children < 15 years of age developed tuberculosis (TB) in 2018, and at least 200,000 children died from TB [1]. Infants, young children, and HIV-positive children are at increased risk for developing TB following infection with *Mycobacterium tuberculosis* [2, 3] and have higher risk of severe TB disease and death than the general adult population [3, 4]. Given the significant burden of TB disease and mortality in young children, implementation of effective child TB contact management (CCM) is an important upstream strategy to prevent TB in children and also provides an opportunity for early case detection and TB treatment initiation. Isoniazid preventive therapy (IPT) has been found to decrease the risk of TB disease in children by 59% [5], and TB screening and IPT in young children have been shown to be highly cost-effective [6]. In 2006, the WHO published the first guidelines for the management of child TB contacts, recommending that national TB control programs conduct contact investigations for TB and offer IPT for child contacts < 5 years of age and for HIV-positive child contacts of any age [7, 8]. Ten years later, in 2016, the WHO added IPT initiation in child contacts < 5 years of age as a formal indicator of TB program performance [9]. Unfortunately while sub-Saharan Africa has the highest childhood TB mortality rates, it has the lowest proportion of child contacts < 5 years of age who received IPT [1, 10]. While many national TB programs have adopted the WHO guidelines, implementation of these guidelines in high TB burden countries remains limited with many countries experiencing operational challenges [11, 12], which ultimately result in poor IPT initiation and completion rates [13].

Lesotho is a lower middle-income sub-Saharan African country [14], with an estimated TB incidence of 611 per 100,000 and HIV prevalence of 25.6%, the highest and second highest rates in the world respectively [1, 15]. Childhood TB in Lesotho is largely managed by TB clinic nurses in health facilities. Nurses provide outpatient care and preventive services and refer complicated cases to district hospitals. Typically each TB clinic is staffed by one to three nurses and one or two lay counselors, who are supported by 20–30 community-based village health workers (VHWs). VHWs are charged with conducting contact investigation in the community. The Ministry of Health (MOH) supplies each health facility with isoniazid 100 mg tablets for the prevention of TB in child contacts as per standard of care; isoniazid suspension is not available in Lesotho.

Childhood TB disease is likely to be underdiagnosed and under-reported as only 4% of reported TB cases in Lesotho are in children < 15 years [1], on the lower end of the expected 10–20% in similar settings [16]. This lower than expected reporting of pediatric cases is most likely due to underdiagnosis of TB in children in Lesotho. In 2011, the Lesotho National TB Program (NTP) adopted the WHO’s recommendations for CCM, but implementation has been limited and the reasons for this wide policy-practice gap in this high TB burden context are unclear. As healthcare providers are gatekeepers of CCM implementation, we explored their attitudes toward TB prevention in children and perceived facilitators and challenges to CCM via qualitative interviews.

## Methods

### Study design, setting and population

We conducted interviews with healthcare providers as part of formative research to inform intervention tool development in our subsequent cluster randomized trial [17]. In collaboration with the Ministry of Health, a single district was chosen for conduct of the trial in order to enhance internal validity and maximize implementation cost efficiency. Berea District was chosen because it was one of the highest TB burden districts and there were no interventions addressing children’s needs in the district. Ten of the 19 public health facilities in Berea district, Lesotho, were randomized to deliver a community-based intervention or standard of care, following stratification by facility type. The remaining nine health facilities were excluded from the sampling frame because of low TB patient case load (on average, < 6 TB patients notified per quarter). Both hospitals (*N* = 2) and health centers (*N* = 8) were included to enhance generalizability of study findings as TB services are provided in both types of facilities in Lesotho. We chose to focus on healthcare providers from the five health facilities that were randomized to deliver the study intervention so as to obtain their feedback on planned intervention components, especially their perceptions of facilitators and challenges to SOC. A sample of 12 healthcare providers was recruited in December 2015, prior to the launch of the cluster randomized trial. The five participating health facilities included one hospital and four health centers. One of the four participating health centers was located in a rural area whereas the other facilities were located in urban areas. During the study period, we enrolled a purposive sample of nine nurses who were the main providers of TB care and all three facility-based lead village health workers (LVHW) who were already employed by the study at that time; all study participants worked in a TB clinic. The main TB providers were targeted by scheduling the interviews ahead of time when they were scheduled to be at the health facility. However, if one of the nurses was unexpectedly unavailable at the scheduled time, we proceeded with the group interview. Nurse participants had standard of care training on TB provided by the Lesotho MOH. LVHW were originally community-based VHWs, who were promoted to LVHW. As community-based VHWs, they underwent the standard MOH training that focused on contact tracing. Inclusion criteria were: nurse or LVHW working in a study site; aged 18 or older; English- or Sesotho-speaking; and capacity and willingness to provide informed consent.

All individuals approached for the study agreed to participate. Four group interviews with nurses and four individual interviews were completed with 12 providers, including nine nurses and three LVHW. The majority (11/12) of participants were female and the mean age was 42 years (range 27–66). Interview duration ranged from 25 to 55 min, with a mean of 37 min.

### Procedures

Four small group interviews were conducted with nurse participants assigned to the same health facility due to logistical reasons such as scheduling, illnesses, unexpected rotations; these were similar to focus group discussions but with a smaller number of participants, capitalizing on group dynamics to capture group norms and experiences. One individual interview was conducted with a nurse at one facility because a group interview was not feasible due to scheduling constraints. Three individual interviews were also conducted with LVHW at three health facilities. Participants were not compensated for participation as per MOH policy but received a snack and fizzy drink.

The study was presented to nurses and LVHW at a district-wide meeting prior to study launch. The trained qualitative interviewer introduced the study to potential participants at each facility as an exploration of their views on CCM and if they were interested, obtained informed consent. Interviews were conducted in English or Sesotho at each participating health facility in a private space on-site. The interview guide consisted of open-ended, exploratory questions that were asked in a non-judgmental and culturally-sensitive way. Respect for participants’ privacy and confidentiality was emphasized in group interviews, and divergent perspectives were encouraged. Interviews were audio-recorded, transcribed verbatim, translated, anonymized and subjected to textual analysis. Study findings were presented and discussed with participants at a district-wide meeting in 2016.

#### Quality assurance

We included several layers of quality assurance monitoring to ensure the integrity of the qualitative interviews. First, the Principal Investigator (YH-M) of this study has considerable experience in developing interview guides and supervising qualitative interviewers. Moreover, she regularly consulted with researchers who have decades of experience with qualitative interviewing (JEM). Second, the interviewer’s experience as a research nurse in other qualitative studies over the prior 4 years provided her with vast experience and expertise in qualitative interviewing. In addition, the interviewer participated in four qualitative interview training sessions for this study. Finally, the interviewer was closely supervised in conducting these interviews and was provided with timely feedback.

#### Domains

We developed an interview guide and pilot tested it with study staff before study implementation. Questions relevant to this analysis addressed training received on childhood TB and CCM and the need for further training; attitudes toward CCM, including community screening, clinic evaluation, and IPT provision; CCM challenges and possible solutions; and stigma in the community (see Supplementary Material).

### Analysis

Study investigators trained in qualitative analysis used thematic analyses as the framework for data inquiry and analysis. We used an iterative analytic process to facilitate a comprehensive understanding of healthcare providers’ perspectives. Initially, we used deductive analysis to identify thematic categories based on our questions and the CCM literature and inductive analysis to generate new codes that emerged from the data. Two study investigators (YH-M, LL) independently reviewed four transcripts (i.e., double-coded) to develop a preliminary coding scheme. We used a “negotiated agreement approach” to ensure consistent interpretation and application of codes, which increases coding reliability [18, 19]. Differences in coding were discussed and consensus reached on how to apply codes. Once consensus on codes was achieved, the final coding scheme was applied to the full set of transcripts. Coding of the remaining transcripts was done independently by the two coders, but they continued to meet regularly to discuss interpretation and application of the codes. Typical quotations are used to illustrate the themes. We used Dedoose (v. 6.2.17), a qualitative software program, for systematic data management and analysis (Socio-Cultural Research Consultants, LLC; Los Angeles, CA) [20].

### Ethical considerations

The study was approved by the Columbia University Irving Medical Center Institutional Review Board (Ref #IRB-AAAN7358) and the National Health Research Ethics Committee in Lesotho (Ref #ID78–2015). The overall study was registered at ClinicalTrials.gov (NCT02662829). All participants provided written informed consent prior to study participation.

## Results

### Facilitators of a successful childhood TB prevention program

Provider understanding of the importance of TB prevention and receiving enhanced provider training on CCM were the major themes regarding a successful childhood TB prevention program. Providers in this study agreed that TB prevention services, including contact tracing, early detection of child TB contacts, and provision of IPT to eligible children, are essential to controlling the TB epidemic in Lesotho. They emphasized the importance of IPT in confronting TB and spoke of the deleterious consequences of TB and the value of the protection that IPT confers to children exposed to TB. Some of the providers believed that CCM is feasible in Lesotho and suggested expanding IPT to *all* household contacts of TB cases and not just to children.

*I see IPT … as a weapon that will protect children.* [LVHW, individual interview].

*It* [IPT] *is good so that they can be safe and protected … all children in their* [household] *should be put on preventive therapy …* [LVHW, individual interview].

*It* [CCM] *prevents spread of TB, eh, because if we are targeting children … it is easier for them to catch the disease … so if they are being targeted, to be screened or to be supplied with isoniazid for 6 months, I think it will help us control the disease and reduce number of TB cases.* [Nurse, individual interview].

*IPT, if it can be given to every contact in the house … everybody that we screened in that house found with no TB should get on IPT.* [LVHW, individual interview].

Providers reported that having enhanced training on CCM, including an emphasis on ruling out TB disease in child contacts, is imperative. They were especially concerned about missing cases of child TB disease and wanted to know more about managing potential side effects from isoniazid (INH).

*I think when we have engaged in trainings, it is then that* … *we are able to attend to side effects … as early as possible. Then we will be equipped with skills … what are the complaints that might come up, those that we are able to identify earlier.* [Nurse, group interview].

*It is difficult to diagnose children, because if you are going to start IPT without any X-ray or sputum samples, we only base ourselves on signs and symptoms … not knowing in depth what is happening to the child.* [Nurse, group interview].

We compared attitudes of nurses and LVHW and did not discern any meaningful differences between the two cadres regarding facilitators of a successful program.

### Challenges to CCM provision and strategies for addressing them

Providers reported multiple challenges to CCM provision and suggested strategies for addressing them. Four themes emerged from the interviews: limited access to care, supply-chain issues, barriers to identification and screening of child contacts, and difficulty with IPT adherence.

#### Limited access to care

Providers reported that caregivers’ ability to bring children to health facilities for CCM was a challenge. In some villages, community-based village health workers (CB-VHW) provide child-related services, such as weighing children. Expanding such community-based services by VHW to include screening and follow-up of child contacts was viewed as potentially advantageous, especially for those experiencing long or difficult journeys to the health facility and in situations where caregivers lack the resources to bring the children for monthly follow-up appointments if IPT is initiated.

*At times it is because these …* [caregivers] *come from far remote areas where transport issues hinder them from making several trips to the clinic.* [LVHW, individual interview].

*They will tell you that they walk long distances, so they cannot carry children from that far.* [LVHW, individual interview].

#### Supply-chain issues

Providers reported stock-outs and shortages of pediatric formulations of INH and vitamin B6, which make it difficult for them to provide IPT to children. Having to adjust adult doses to pediatric ones can be challenging as the INH pills have to be cut in two or four depending on the child’s weight. They indicated that availability of a reliable drug supply would facilitate IPT implementation.

*The challenges are … inconsistent supply of appropriate drugs.* [Nurse, individual interview].

*It becomes very difficult for us. It* [INH] *takes a long time to be replaced. Also, to reduce the adult dose for children is so difficult.* [Nurse, group interview].

#### Identification and screening of child contacts

Providers believed that identifying and screening children for TB was the main challenge and that once children come to the clinic, providing IPT is feasible. Community education and the CB-VHW were seen as pivotal to supporting caregivers to bring their children to the clinic for evaluation.

*The problem is contacting or tracing of contacts. But those who have been inside here, there are no problems.* [Nurse, group interview].

*I usually ask the parent to come so that we have* a *one-on-one health talk so that I explain clearly the benefits of getting into the preventive therapy and the risks of denying this therapy. So with persuasion, we see some do bring their children, but some are just hard-hearted and they don’t.* [Nurse, individual interview].

In cases where caregivers do not bring in the child contacts for evaluation, CB-VHW are deployed to assist, and when they have difficulty tracing patients, the village chief is informed so that he can assist in finding them.

*We use our CB-VHW to help us bring them. Those are the ones who assist us most of the time.* [Nurse, group interview].

*If they* [CB-VHW] *are unable to find and bring them, then we inform the village chief that we have a TB patient in his village and that we need* [to assess] *their children.* [Nurse, group interview].

Nurses and LVHW emphasized the need for health education as they believed that community members do not recognize the importance of preventing TB in child contacts, including the need for infection control in situations where a presumptive or confirmed TB case has been identified in the household. Providers suggested that community-based health education can reach more people and have a greater impact, especially by strengthening the role of CB-VHW to educate families in the community.

*We have to educate them … so that they understand why, because then if while a child is on IPT, it means it is a long period of time.* [Nurse, group interview].

*There is a need to go out to schools, during public gatherings, wherever we are able to go and reach out to provide health education.* [Nurse, group interview].

*We also ask the CB-VHW to give the health education at the village so that the parent will end up understanding that it is important to have the children on IPT.* [Nurse, group interview].

#### IPT adherence

Providers indicated that IPT adherence was another major challenge, possibly because of fear of the drug’s side effects such as a severe rash. To tackle this issue, they suggested continuous enhancement of health literacy for caregivers about the importance of adherence and the possibility of side effects from IPT.

*The challenge is the child cannot come regularly. She comes once, skips a month. When you follow the child again, the child reappears so there is drug interruption.* [Nurse, individual interview].

*The challenge is that we find later after they have missed doses of IPT because of experiencing side effects.* [Nurse, group interview].

When we compared attitudes of nurses and LVHW regarding challenges to CCM, LVHW were more focused on issues of access-to-care whereas nurses were more concerned about supply-chain issues.

### Perceived stigma

Stigma was not reported as a CCM challenge. When providers were specifically questioned about the role that stigma plays, they indicated that stigma related to TB prevention is generally not perceived as an issue in the community. However, some providers acknowledged that stigma may play a role in some situations where caregivers did not bring children to the clinic. Others believed that lack of knowledge, not stigma, is the reason that caregivers did not bring children to the clinic. Providers felt that community health education campaigns could help to increase knowledge regarding TB and dispel any possible stigma.

*TB these days is no longer stigmatized; everybody is willing … so they do not have a problem if you want to screen them in the community.* [Nurse, group interview].

*Not stigma as such, lack of knowledge maybe … I don’t think stigma is still a problem.* [Nurse, individual interview].

*It* [IPT] *won’t put them at risk of stigma because they would have accepted that their children should be initiated* [on IPT]. [LVHW, individual interview].

*Those who are bringing their children do not have the problem* [stigma], *but I could see or sense that these ones who don’t want to bring in their children, they think in some way of stigma.* [Nurse, individual interview].

## Discussion

In this manuscript, we report on healthcare providers’ perceptions of facilitators and challenges. These data were collected as part of formative research in preparation for our subsequent cluster randomized trial. A limited number of studies have explored provider attitudes regarding TB prevention in children in a high TB burden setting in-depth [13]. In this qualitative study, we found interest and commitment among healthcare providers in Lesotho to improve CCM. Providers reported a need to have enhanced training on CCM, with an emphasis on ruling out TB disease among child contacts and advocated for strong community health education efforts as they reported that community members do not currently recognize the importance of preventing TB in child contacts. Such efforts would enable caregivers of TB child contacts to appreciate the importance of preventing TB in such children and the role that TB prevention can play in their children’s health and well-being.

**Fig. 1:**
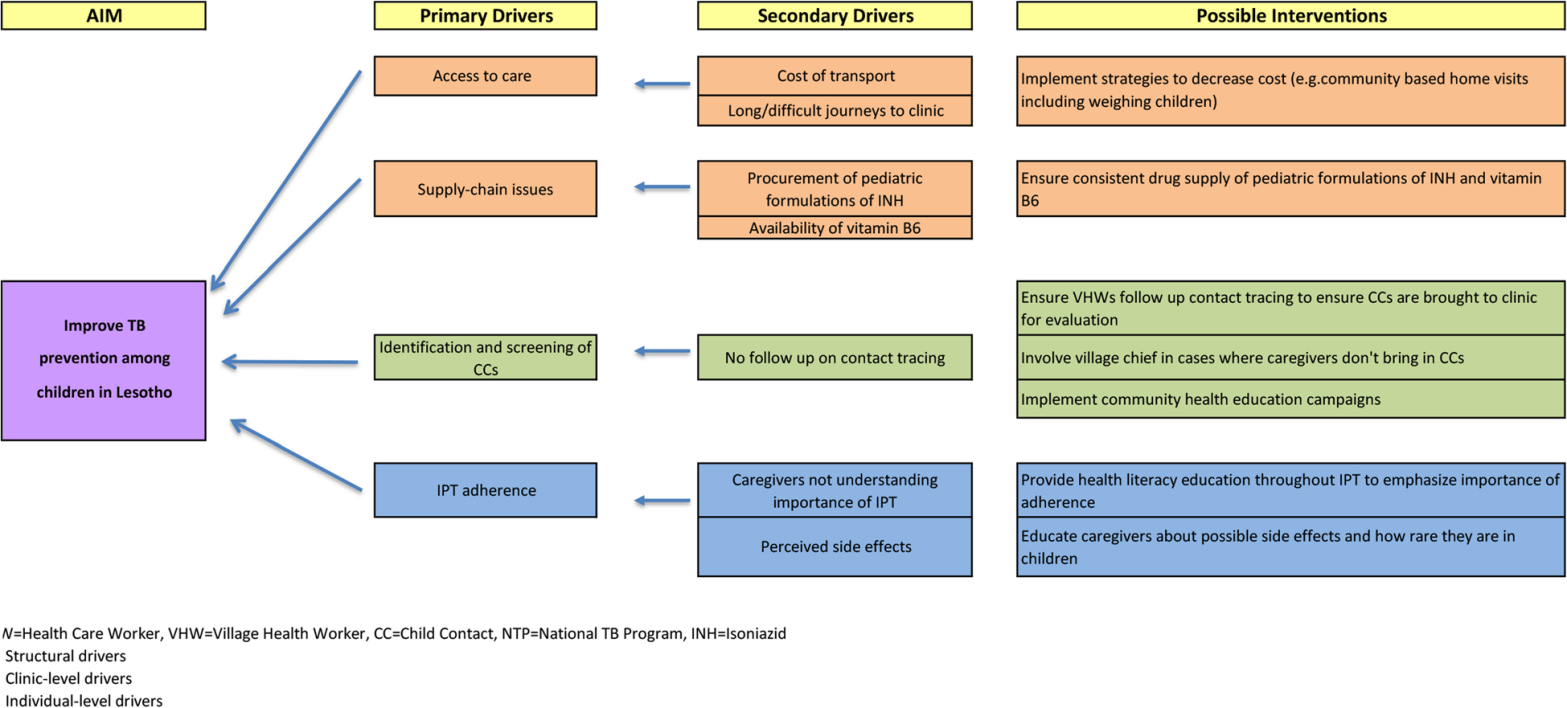
Driver Diagram for CCM in Lesotho

The challenges identified in this study suggest the primary drivers that need to be addressed to achieve successful TB prevention in children in Lesotho, summarized in the driver diagram in Fig. 1. We identified that prohibitive cost of transport and long and difficult journeys to the clinic were structural challenges to CCM. This is similar to findings from other studies that employed qualitative methods and reported costs and transportation challenges as major barriers [21–26]. Providers in our study suggested strategies to decrease costs to caregivers, such as home visits that include weighing children so that INH dosages can be adjusted according to the child’s weight, precluding the need for caregivers to bring children to the clinic every month. Another structural challenge to IPT delivery was the lack of reliable supply of pediatric formulation of INH and vitamin B6, an issue that has been reported in prior studies [21, 27, 28]. Although according to the Lesotho NTP guidelines, vitamin B6 is recommended to be given with INH, nurses are encouraged to prescribe INH even in the case of vitamin B6 shortage as peripheral neuropathy is uncommon in young children [29]. It is unclear whether the INH stock-outs and shortages are due to procurement problems or inefficient distribution of the supply of the drug to health facilities. It is also possible that the shortage may be due to inaccurate forecasting of the required doses of INH and vitamin B6 that are needed.

Identification and screening of child contacts was found to be a clinic-level challenge that affects the success of TB prevention efforts. Lack of community contact tracing follow-up contributed to this challenge. These challenges likely arose from gaps in caregiver education about the importance of TB prevention, which has been reported in other studies [21, 23, 26, 27, 30, 31]. Providers recommended that caregivers be educated in the community by CB-VHW so that they will be convinced of the need to have their children evaluated. Additionally, the Lesotho TB program has had some success involving the village chiefs in motivating reluctant caregivers to take their children for evaluation at the health facilities.

IPT adherence was an individual-level barrier to CCM, with providers reporting that caregivers may not fully understand the importance of adherence and worry about possible side effects from IPT. Caregiver concerns about side effects have been reported in studies from India and Indonesia [22–24, 27]. Providers suggested that health literacy education about the importance of adherence with IPT and possible side effects might encourage caregivers to more promptly present at the health facilities if their children demonstrate either of these. This might also alleviate the failure to complete IPT course by children that may be due to side effects experienced by the children.

While providers did not perceive stigma as an important issue in the community as related to TB and IPT when specifically questioned, some acknowledged that it may play a role in situations where caregivers failed to bring children to the clinic. Skinner et al. similarly found that providers felt that TB was so widely prevalent that it could no longer be stigmatized. The role of stigma needs to be further explored among caregivers of child TB contacts [31].

Our qualitative evaluation had several limitations. Given the small sample of providers drawn from a small number of health facilities, the findings may not reflect the experiences of all healthcare providers engaged in public-sector TB care in Lesotho, limiting our ability to draw definitive conclusions about CCM and IPT. We conducted provider interviews at five health facilities in a combination of small group and individual interviews and were limited to those providers who worked at those facilities and were available on the interview day. Our sample is relatively homogenous --study participants are well positioned to reflect on study topics, work for the MOH and all were exposed to the basic MOH training on TB. The interviewer’s familiarity with the healthcare providers in study facilities may have led to either more honest discussions or socially desirable responses.

Nevertheless, the study also had several strengths. Our qualitative study explored provider attitudes in-depth, thereby providing rich data collected in a rigorous way as we included several layers of quality assurance monitoring to ensure the integrity of the qualitative interviews. Interpretations were contextualized with illustrative quotes to enhance their applicability and relevance to similar high TB burden settings. Our sample included both nurses and LVHW, who were able to provide different perspectives on challenges, with LVHW focusing on issues of access-to-care and nurses expressing concerns about supply-chain issues. Few studies conducted to date employed qualitative methods to explore in-depth provider attitudes toward TB prevention in high TB burden settings, and only two of these qualitative evaluations were conducted in sub-Saharan Africa [21, 31].

## Conclusions

Despite a decade-long recommendation from the WHO to conduct CCM in high TB burden settings, its implementation in Lesotho has been limited. However, we found interest and commitment among TB providers in our study to improve CCM. Challenges identified by providers were at multiple levels -- structural, clinic, and individual. Given the significant burden of TB morbidity and mortality in young children and the recent requirement by the WHO to report IPT initiation in child contacts, prioritization of CCM is imperative and should include enhanced provider training on childhood TB and CCM and mentorship as well as strategies to eliminate challenges. Strategies that enable a more efficient CCM delivery include creating standardized tools that facilitate the implementation, tracking, and monitoring of child TB contact management coupled with guidance and mentorship from the district health management team. To tackle access to care challenges, we propose delivering intensive community health education, conducting community screening more efficiently using standardized tools, and facilitating access to services in the community.

## Supplementary information

**Additional file 1.**

## Data Availability

All relevant data collected during the current study will be available from the corresponding author on reasonable request.

## Abbreviations

CCM: Child contact management
INH: Isoniazid
IPT: Isoniazid preventive therapy
LVHW: Lead village health worker
MOH: Ministry of Health
NTP: National TB program
TB: Tuberculosis
VHW: Village health worker
WHO: World Health Organization
