# Supplementary material for: Provider attitudes about childhood tuberculosis prevention in Lesotho: a qualitative study
Source: BMC Health Serv Res. 2020 May 25;20:461. doi: 10.1186/s12913-020-05324-0 (PMC7249694; doi:10.1186/s12913-020-05324-0)
Supplement: Supplementary file 1 — Additional file 1. [file 12913_2020_5324_MOESM1_ESM.pdf]

PROVIDER INTERVIEW GUIDE

EXPLORATORY PRE-INTERVENTION

#	Primary Question	Secondary Question
**1.Introduction and general information**		
I would like to start off by talking about your work in this TB clinic.		
1.1	Please tell me about your work at this facility.	• What are your responsibilities?<br>• How long have you worked here?<br>• Has your position changed since you started working here? How?
1.2	Can you tell me about the patients you care for?	• Do you screen patients for TB every day or on certain days of the week?<br>− How about providing TB treatment?<br>• Do you screen children for TB in this clinic? **IF YES**, how often?
1.3	Can you tell me about a 'typical' day in your clinic?	
1.4	What do you think about the TB situation in this community?	
1.5	How do you feel about providing services for persons with TB?	• How do you feel about providing TB services to children?
**2. Training needs**		
Now let's talk about training you received and training needs.		
2.1	What training have you received in TB?	• Tell me about your in/formal education/on-the-job training or continuing education.<br>• When were you trained/by whom?
2.2	Have you received any training on TB contact screening?	• How well prepared do you feel to deal with TB contact tracing?
2.3	Have you received any training on IPT provision?	• How well prepared do you feel to deal with IPT?
2.4	What do you think about the training you have received?	• How satisfied or dissatisfied are you with the training you received?<br>• Do you want to have access to more information? **IF YES**, what content would be helpful? How would you like it delivered?
2.5	How does your program ensure health workers are trained adequately on TB?	
2.6	How often do staff turnover or change in this facility?	
**3.Attitudes towards TB prevention**		
Now I would like to talk with you about TB prevention		
3.1	What are your thoughts about screening child contacts of TB patients?	
3.2	What about starting child contacts on IPT?	
3.3	What are your thoughts about testing ALL child contacts for TB?	
3.4	In your experience, what are the benefits of providing TB prevention services?	
3.5	In your experience, what are the challenges of providing TB prevention services?	

3.6	Given your experience, what are other challenges with implementation of TB prevention services?	• Have you encountered challenges with IPT, e.g., patient issues, inadequate resources, dissemination problems?
**4.Implementation**		
Now let's talk about the IPT tools that were recently introduced in Berea District. These tools include a child contact tracing register and a facility-based IPT card.		
4.1	How have TB prevention services changed or not changed in the last 3-4 months?	
4.2	What do you do if a patient refuses to bring in their child contacts for TB screening?	
4.3	What kinds of problems come up when treating child contacts?	• How do you overcome these problems?
4.4	What kinds of challenges has delivery of TB prevention services presented for health care workers?	• How has that affected your workload? **PROBE for** staff burden<br>• What do you think are the biggest challenges?<br>• How can these challenges be overcome?
4.5	What about you? Can you describe some of the problems you have encountered with providing TB prevention services?	• How would you fix these problems?
4.6	Do you think that health care workers believe that TB prevention services will work?	• Why or why not?
4.7	If you had the opportunity, what would you change about the way TB prevention services are provided in your facility/clinic?	• Can you think of any ways to improve the delivery of health services in relation to TB prevention?
**5.Stigma**		
We are getting very close to the end of our interview. I just have a few more questions.		
5.1	Have parents been bringing in their child contacts to the clinic for TB screening?	• Why or why not?
5.2	Would parents be more likely to have their child contacts screened in the community?	• Why or why not?
5.3	Do you think that providing IPT to child contacts might expose them or their families to stigma?	• **IF YES**, in what ways?
**6. Intervention components**		
Before we end I'd like to get your feedback about additional intervention components.		
6.1	What do you think about VHW screening of child contacts in the community?	• What are the main benefits to that?<br>• What are the main barriers?<br>• How can we strengthen this component?
6.2	Do you feel that creating job tools such as a clinical algorithm, would be helpful for delivering TB prevention services?	• **IF YES**, what content would be helpful? How would you like it delivered?
6.3	Do you feel that creating patient education tools such as a flipchart, would be helpful for delivering TB prevention services?	• **IF YES**, what content would be helpful? How would you like it delivered?<br>• What kind of information would you like to see in the flipchart?<br>• Is there another format in which this information can be delivered?
**7.Other**		
We are done but I would just like to know a little about you.		
7.1	Please tell me how old you are.	
7.2	What is the highest level of education you	

	completed?	
7.3	How long have you been employed in this position?	
7.4	Have I missed anything? Is there something you would like to add?	
